# Neoadjuvant Therapy in Locally Advanced Rectal Cancer—What Result Should We Expect?

**DOI:** 10.3390/medicina62040793

**Published:** 2026-04-21

**Authors:** Roxana-Elena Stefan, Adrian Constantin, Daniela Dinu, Florin Achim, Alexandru Rotariu, Florin Grama, Horia-Dan Liscu, Lucian Iordache, Dragos-Viorel Scripcariu, Anthony Rasuceanu, Silviu Constantinoiu, Dragos Predescu

**Affiliations:** 1 Doctoral School, Carol Davila University of Medicine and Pharmacy, 050474 Bucharest, Romania; roxana-elena.stefan@drd.umfcd.ro; 2 Faculty of Medicine, Carol Davila University of Medicine and Pharmacy, 050474 Bucharest, Romania; dradiconstantin@yahoo.com (A.C.); dagnypedersen@yahoo.com (D.D.); alexandru.rotariu95@yahoo.com (A.R.); florin_gramma@yahoo.com (F.G.); horia.liscu@umfcd.ro (H.-D.L.); ionut-lucian.antone-iordache@drd.umfcd.ro (L.I.); rasuceanu.anthony@gmail.com (A.R.); silviuconstantinoiu@gmail.com (S.C.); drpredescu@yahoo.com (D.P.); 3 Department of General and Esophageal Surgery, Sf. Maria Clinical Hospital, 011192 Bucharest, Romania; 4 Department of General Surgery, Coltea Clinical Hospital, 030167 Bucharest, Romania; 5 Department of Radiotherapy, Emergency University Hospital, 050098 Bucharest, Romania; 6 Department of Radiotherapy, Coltea Clinical Hospital, 030167 Bucharest, Romania; 7 Faculty of Medicine, Grigore T. Popa University of Medicine and Pharmacy, 700115 Iasi, Romania; 8 First Oncological Surgery Unit, Regional Institute of Oncology, 700483 Iasi, Romania

**Keywords:** locally advanced rectal cancer, total neoadjuvant therapy, tumor regression grade, systemic inflammatory markers, carcinoembryonic antigen, lymphocyte count, chemoradiotherapy

## Abstract

*Background and Objectives*: Neoadjuvant chemoradiotherapy is a key component of the treatment strategy for locally advanced rectal cancer (LARC), both through its direct impact on oncological prognosis and by increasing the likelihood of sphincter-preserving surgery. Oncological prognosis improves dramatically following a complete pathological response to neoadjuvant therapy. Identifying predictors of response to neoadjuvant therapy has been a challenge over the past two decades, and these factors have not been fully identified. This study aimed to analyze the clinical, biological, and therapeutic factors associated with tumor response following neoadjuvant therapy in patients with locally advanced rectal cancer, with the aim of identifying independent predictors of the absence of a complete pathological response and optimizing personalized treatment strategies. *Materials and Methods*: This retrospective study included a cohort of 122 patients (81 men and 41 women), with a mean age of 63.5 years, diagnosed with locally advanced rectal cancer at two centers with expertise in colorectal surgery between January 2018 and December 2023. Patients received neoadjuvant treatment in two regimens: long-course chemoradiotherapy with oral radiosensitizing chemotherapy (82 patients) and total neoadjuvant therapy consisting of chemoradiotherapy followed by consolidation chemotherapy (40 patients). A series of clinical, biological, and therapeutic variables was analyzed for their association with pathological responses. *Results*: According to the Ryan score, the overall complete response rate following neoadjuvant therapy was 17.2%. pCR was observed more frequently in patients treated with total neoadjuvant therapy than in those treated with standard chemoradiotherapy. Elevated pre-treatment CEA levels were independently associated with a higher risk of unfavorable tumor response. The radiation dose and interval between completion of radiotherapy and surgery were significantly associated with tumor regression. *Conclusions*: These results underscore the importance of personalizing neoadjuvant therapy to improve cancer prognosis. Furthermore, optimizing tumor regression could lead to the potential expansion of sphincter-preserving resection techniques, which would have a direct and significant impact on the quality of life of these patients.

## 1. Introduction

Colorectal cancer is a major public health problem worldwide. According to GLOBOCAN 2022 estimates, this disease is the third most common type of cancer worldwide and one of the leading causes of cancer-related mortality [1]. Although the incidence of the disease is traditionally associated with advanced age, recent epidemiological studies have highlighted a steady increase in the incidence of rectal cancer among patients under 50 years of age, a phenomenon described in the literature as “early-onset colorectal cancer” [2,3]. This emerging trend raises significant concerns regarding the etiopathogenic mechanisms involved and optimal therapeutic management strategies.

Locally advanced rectal cancer (LARC) is defined by the presence of tumors that extend beyond the muscularis propria of the rectal wall (stages T3–T4) and/or involvement of regional lymph nodes (N+) [4]. Approximately 70–80% of newly diagnosed cases fall into this clinical category, characterized by an increased risk of local recurrence and distant metastasis [5]. In addition, a significant proportion of patients diagnosed with LARC are under 50 years of age, which gives this condition considerable clinical and socioeconomic relevance.

In recent decades, the therapeutic management of locally advanced rectal cancer has evolved significantly with the introduction of multimodal treatment strategies. The current standard of care includes neoadjuvant chemoradiotherapy followed by radical rectal resection with total mesorectal excision (TME), a surgical technique first described by Heald et al., and is associated with a significant reduction in local recurrence [6]. In certain situations, surgical treatment is followed by adjuvant chemotherapy, depending on the tumor’s pathological characteristics.

Two main strategies are used in neoadjuvant therapy: long-course chemoradiotherapy (LCRT) and short-course radiotherapy (SCRT). LCRT involves administering a total radiation dose of approximately 45–50.4 Gy over 5–6 weeks, concurrently with radiosensitizing chemotherapy, most commonly fluorouracil or capecitabine [7]. Numerous clinical studies have demonstrated that this therapeutic strategy reduces the local recurrence rate, increases the likelihood of achieving negative circumferential resection margins, and facilitates sphincter preservation in cases of low rectal tumors [8,9,10].

Alternatively, SCRT involves administering a total dose of 25 Gy in five consecutive fractions, followed by surgery within a short period of time [11]. Randomized studies, including the Polish trials and the Trans-Tasman Radiation Oncology Group study, have demonstrated comparable outcomes between SCRT and LCRT in terms of local disease control and overall survival [12,13]. However, some analyses have suggested that SCRT may be associated with lower rates of complete pathological response and, in certain contexts, a higher incidence of positive circumferential resection margins than LCRT [4,5]. Consequently, long-course chemoradiotherapy remains the preferred strategy according to most international clinical guidelines [14].

Tumor response to neoadjuvant therapy is a major prognostic factor for locally advanced rectal cancer. It is assessed through clinical examination, endoscopic evaluation, and pelvic magnetic resonance imaging (MRI), typically performed approximately six weeks after completion of neoadjuvant treatment [15]. A clinical complete response (cCR) is reported in approximately 10–40% of patients, while pathological complete response (pCR), defined as the absence of viable tumor cells in the resection specimen (ypT0N0M0), is observed in a smaller percentage of patients [16].

Despite significant therapeutic advances in the management of locally advanced rectal cancer, tumor response to neoadjuvant therapy remains characterized by considerable interindividual heterogeneity. This variability reflects the biological complexity of rectal tumors and the interaction between the tumor’s intrinsic molecular characteristics, tumor microenvironment, and the host’s systemic response. Consequently, while some patients may achieve major tumor regression or even a complete pathological response after neoadjuvant therapy, others exhibit a limited response, associated with residual disease and an increased risk of recurrence.

The prognostic significance of pCR has been well documented. Patients who achieve a pathological complete response have significantly lower rates of local recurrence and superior outcomes in terms of disease-free survival than those with a partial or no tumor response [17]. In this context, tumor regression grade (TRG) was introduced as a standardized method for histopathological assessment of tumor response following neoadjuvant therapy. Over time, several classification systems have been proposed, including the Mandard [18], Dworak [19], Memorial Sloan Kettering Cancer Center, and Ryan classifications, the latter of which is currently adopted in the American Joint Committee on Cancer (AJCC) staging system [20].

Over the past decade, advances in therapeutic strategies have led to the development of the concept of total neoadjuvant therapy (TNT), which integrates the administration of multi-agent systemic chemotherapy in sequence with neoadjuvant chemoradiotherapy before surgery. This approach aims to intensify early systemic treatment to eradicate micrometastases and increase the rate of complete pathological response. Major clinical trials, such as RAPIDO and PRODIGE 23, have demonstrated the superiority of this strategy over conventional approaches in terms of disease control and disease-free survival [21,22].

Despite significant therapeutic advances, the response to neoadjuvant therapy remains heterogeneous and difficult to predict. Therefore, identifying robust predictors of tumor regression represents a major area of research, with direct implications for the individualization of treatment. Numerous studies have investigated potential predictors of achieving a complete pathological response, including low levels of carcinoembryonic antigen (CEA), small tumor size, less advanced lymph node stage, histological grade of differentiation, low tumor location, and limited circumferential tumor extension [23,24,25,26]. Additionally, systemic inflammatory parameters, such as the neutrophil-to-lymphocyte ratio (NLR), have been proposed as potential biomarkers of therapeutic response [27].

At the same time, certain treatment-related factors have been associated with the likelihood of achieving a complete response, including the type of concurrent chemotherapy, the total dose of radiation therapy, and the interval between the completion of chemoradiotherapy and surgery [28]. The duration of the radiotherapy–surgery interval has been extensively investigated, with available data suggesting that extending this interval beyond 6–8 weeks may be associated with increased rates of complete pathological response [29].

In this context, identifying clinical and biological markers capable of predicting tumor response to neoadjuvant therapy is crucial for optimizing treatment strategies and developing personalized approaches for the management of locally advanced rectal cancer.

### Aim of the Study

The study examined factors influencing tumor response to neoadjuvant therapy in locally advanced rectal cancer to identify predictors of incomplete pathological response.

## 2. Materials and Methods

This retrospective study included 122 patients with LARC hospitalized and treated at two surgical tertiary centers with expertise in colorectal surgery between 2018 and 2023. The aim of this study was to identify the factors leading to a complete pathological response after neoadjuvant therapy, as well as those associated with a near-complete response or those who did not achieve a response following neoadjuvant therapy.

The inclusion criteria were age > 18 years, locally advanced rectal cancer (lower/middle/upper rectum) (stage II-III), after completion of neoadjuvant chemoradiotherapy with oral Capecitabine (LRCT) or total neoadjuvant therapy (TNT), consisting of chemoradiotherapy followed by consolidation chemotherapy with CapeOx/FOLFOX, followed by surgical rectal resection with TME. Patients included in the study were followed for a minimum of 12 months.

The exclusion criteria were as follows: patients with early-stage rectal cancer, patients with metastases, and patients who underwent a “watch-and-wait” follow-up after radiation therapy.

Complete staging was performed following colonoscopy with biopsy. The oncological workup included contrast-enhanced CT of the chest, abdomen, and pelvis. The extent of local disease was assessed using contrast-enhanced pelvic MRI.

Patients underwent either long-course chemoradiotherapy, consisting of five sessions per week of external beam radiotherapy with conventional fractionation, over 5–6 weeks of radiotherapy, reaching a total dose of 45–50.4 Gy, concurrently with capecitabine 825 mg/m^2^ twice daily, or long-course chemoradiotherapy followed by consolidation chemotherapy (TNT). The decision to administer TNT was based on a preoperative oncological risk assessment, performed by a multidisciplinary committee and primarily based on MRI imaging characteristics. TNT was preferred in patients with high-risk factors for local or systemic recurrence, such as CT4 tumors, significant extramural invasion, questionable circumferential margin (CRM < 1 mm), extramural venous invasion (positive EMVI), extensive lymph node involvement (N2), or large tumors. In these situations, intensification of systemic therapy prior to surgery is aimed at increasing the pathological response rate, improving early systemic control, and reducing the risk of micrometastases.

In the absence of these major risk factors, patients were referred for standard neoadjuvant chemoradiotherapy. None of the 122 patients experienced interruptions longer than 3 days due to acute adverse reactions during radiotherapy. Patients who received consolidation chemotherapy did not experience acute adverse reactions greater than grade 3. Patients at risk of pre-irradiation obstruction (circumferential tumors occupying > 50% of the lumen) underwent pre-treatment laparoscopic colostomy. Six weeks after neoadjuvant therapy, patients were evaluated via clinical examination, MRI with a structured report, and colonoscopy. Postoperatively, patients were stratified based on pathological response, identifying complete or near-complete tumor regression, partial response, or absence of tumor response.

Subsequent surgical resection was performed using both open and laparoscopic approaches, approximately 6–8 weeks after radiation therapy. The procedures consisted of low or ultralow anterior rectal resection with classic or delayed coloanal anastomosis, or abdominoperineal resection.

Assessment of response to neoadjuvant therapy was performed using a TRG system recommended by the American Joint Committee on Cancer (AJCC), specifically the modified Ryan score.

The modified Ryan score includes the following categories: 0-No viable cancer cells (complete response); 1—Single cells or rare small groups of cancer cells (near complete response); 2—Residual cancer with evident tumor regression, but more than single cells or rare small groups of cancer cells (partial response); 3—Extensive residual cancer with no evident tumor regression (poor or no response) [11].

All patients signed an informed consent form authorizing the use of their data and participation in research activities.

The study was conducted in accordance with the Declaration of Helsinki and approved by the Ethical Committee of the Sf. Maria Bucharest (approval form no. 12287/23 May 2023) and Clinical Hospital Coltea (no. 42/13 February 2025).

### Statistical Methods

For statistical analysis, we used JASP version 0.95.1 (JASP Team, Amsterdam, The Netherlands) and R version 4.5.2 (R Core Team), RStudio 2026.1.0.392 (Vienna, Austria). Continuous variables were presented as mean, standard deviation (SD), median, and interquartile range (IQR), while categorical variables were presented as numbers and percentages. The normality of continuous variables was assessed using the Shapiro–Wilk test. We investigated differences between the good response (Ryan 0, 1) and poor response (Ryan 2, 3) using several modalities. In the case of continuous variables, we used Student’s *t*-tests/Mann–Whitney U tests to check for differences in means between response groups for: age at diagnosis, longitudinal tumor dimension, length of radiotherapy (in days), length from the end of radiotherapy to surgery (in days), lymphocyte, neutrophil counts, neutrophil-to-lymphocyte ration (NLR), carcinoembryonic antigen (CEA), length to anal verge. For categorical variables, we used univariate logistic regression: radiation dose (split into <46 Gy categories and >46 Gy categories), type of chemotherapy, type of surgery (classical vs. laparoscopic surgery), and obesity (yes/no). As this observational cohort was exploratory in nature, we did not adjust for multiple comparisons. However, we performed an exploratory post hoc multiple logistic regression model to investigate whether the variables found to be significant in the univariate analysis retained their significance when adjusted for the others. Collinearity was assessed by calculating the tolerance and variation inflation factor (VIF) with threshold values for significant collinearity of >0.2 and <5, respectively. We used contingency tables and Fisher’s exact test to check for associations between the type of chemotherapy and tumor and nodal response.

## 3. Results

After applying the inclusion/exclusion criteria, 122 patients with LARC were included in the study. The cohort consisted predominantly of male patients (66.4%); the mean age of the patients was 63.5 ± 9.4 years, ranging from 30 to 84 years, with the majority having tumors located in the lower rectum (72.1%) and an overwhelming proportion of G2 adenocarcinomas (87.7%), not otherwise specified (NOS). Due to the small number of mucinous cases, a robust statistical analysis of the influence of histological subtype on tumor regression was not possible.

Most patients were diagnosed with stage III. The overall complete response rate, as assessed by the Ryan score, was 17.2%, 6.1% for patients treated with Capecitabine-based chemoradiotherapy vs. 40% for the TNT group (Appendix A Table A1).

The demographic data of the cohort are presented in Table 1.

In order to see if there are differences in the values of several continuous variables (age at diagnosis, longitudinal tumor dimension, length of radiotherapy, length from the end of radiotherapy to surgery, lymphocyte, neutrophil counts, NLR, CEA, length to anal verge) between patients who had a good response to chemoradiotherapy (Ryan 0 and 1) and patients who had a poor response (Ryan 2 and 3) independent-sample tests were performed. The results are presented in Table 2.

The Shapiro–Wilk test was used for normality assessment, and age at diagnosis was the only normally distributed variable. Therefore, Student’s *t*-test was used for age at diagnosis, and the Mann–Whitney U test was used for the rest of the variables. The results are presented in Table 3. The good response group had significantly lower serum CEA levels (*p* = 0.001), significantly higher lymphocyte counts (and conversely significantly lower NLRs, *p* = 0.013), and a longer interval between radiotherapy and surgery (*p* < 0.001).

We investigated whether several binary variables (radiation dose, chemotherapy type, surgery type, and obesity) influenced the response to chemoradiotherapy. We employed univariate logistic regression, using binary variables as predictors of good and poor responses. The results are summarized in Table 4. Higher RT doses and total neoadjuvant treatment were associated with better response (*p* = 0.015; *p* < 0.001), whereas the type of surgery and obesity had no influence on the Ryan score (*p* = 0.546; *p* = 0.665).

Neoadjuvant treatment consisted of Capecitabine-based chemoradiotherapy (CRT) in 67.2% of cases and total neoadjuvant therapy (TNT), which in our study consisted of long-course chemoradiotherapy followed by consolidation chemotherapy (CapeOX/FOLFOX) over 12–16 weeks in 32.8% of cases. Radiotherapy doses exceeding 46 Gy were administered to 83.6% of patients. The radiotherapy doses used in LCRT typically range between 45 and 50.4 Gy, in accordance with clinical standards for neoadjuvant chemoradiotherapy for rectal cancer. In our analysis, the 46 Gy threshold was chosen to distinguish regimens at the lower end of the standard range from those with the highest doses within this range, allowing the evaluation of tumor response differences within clinically accepted treatment regimens.

A pathological complete response, as assessed using the TRG system recommended by AJCC, was achieved in 21 patients (Ryan 0 = 17.2%), while 24 patients achieved a near-complete response (Ryan 1), with the remaining 77 patients presenting residual tumor (Ryan 2–3). Lymph node sterilization (ypN0) was observed in 72.1% of cases.

To analyze the association of tumor and nodal stage at diagnosis and Ryan score, we employed two contingency tables (Appendix A Table A2 and Table A3) and used Fisher’s exact test, with no significant results (*p* = 0.17 for the tumor stage and *p* = 0.36 for the nodal stage).

We employed Fisher’s exact test (contingency tables in Appendix A Table A4 and Table A5) to assess whether, after neoadjuvant treatment, there were any significant differences in the tumor and nodal staging depending on the chemotherapy regimen used. Notably, the TNT group presented significantly lower pathological tumor stages (*p* < 0.001) and nodal stages (*p* = 0.019) than the Capecitabine group.

We built an exploratory post hoc multiple logistic regression model (Table 5) for all the predictors shown to be significant in association with the Ryan score, to determine whether they retained their effect when adjusted for the others. While the model classified poor response (Ryan score of 2 or 3) as an event, the rule of approximately 10 events per predictor was respected. McFadden R^2^ indicated a good model fit (0.417). However, this should be interpreted with great caution, as the model, considering the design, suffers from optimism bias, was not internally or externally validated, and is a post hoc test. All the predictors retained their significance, except for NLR, which likely lost significance because it was derived from lymphocyte count (itself a significant predictor in the multiple model).

## 4. Discussion

Tumor regression following neoadjuvant therapy in LARC is a complex biological process determined by the interaction between tumor characteristics, host immune competence, and treatment intensity. In the present study, both systemic biomarkers and therapeutic variables independently influenced tumor regression, suggesting that the response to treatment reflects not only cytotoxic exposure but also tumor interactions.

pCR is a key factor in achieving favorable long-term oncological outcomes in rectal cancer. In our cohort, pCR was achieved in 17.2% of patients, a rate consistent with previously reported results in the literature, for long-course chemoradiotherapy, where rates generally range between 10 and 30% [11,13,30]. The prognostic significance of pCR is well documented; patients who achieve this type of response have significantly lower rates of local recurrence and superior DFS than those without a complete response [14,16,31].

There is ongoing debate in the literature regarding the effectiveness of different radiotherapy strategies on pCR rates. Some studies have shown that pCR rates are comparable between short-course and long-course radiotherapy [11,12], while other analyses suggest that opting for LCRT may lead to higher tumor regression rates [32]. Currently, several clinical trials have evaluated the feasibility of a conservative approach in patients with major tumor response following neoadjuvant therapy, such as local excision or “watch-and-wait” strategies [23,28,33]. The results of these studies have demonstrated that oncological control may be comparable to that achieved through radical resection with TME in carefully selected patients. Our study does not allow a direct evaluation of these strategies, as all included patients were treated with LCRT, without the inclusion of SCRT in the treatment regimen.

TNT can enhance tumor regression by increasing cytotoxic exposure, treating micrometastatic disease early, and modulating systemic immune responses. Early administration of systemic chemotherapy can reduce tumor volume and alter the tumor microenvironment, thereby facilitating an improved response to radiotherapy.

Several studies have evaluated the role of TNT in LARC [21,22,34]. The main randomized trials comparing TNT with standard neoadjuvant therapy are RAPIDO and PRODIGE-23, which demonstrated an increase in pCR rates and improved DFS without a significant increase in overall toxicity [17,18]. Available data suggest that administering systemic chemotherapy in the neoadjuvant sequence, rather than the adjuvant sequence, may lead to superior oncological outcomes and a reduction in toxicity associated with adjuvant therapy [35]. In our study, the independent association between TNT and favorable tumor regression confirms these observations and supports the concept that intensifying systemic treatment contributes to more effective tumor eradication than conventional chemoradiotherapy.

Achieving pCR has been correlated with the administered radiotherapy dose in numerous studies [14,32,35]. The NCCN guidelines recommend a standard dose of approximately 45 Gy for resectable tumors, with the option of administering an additional boost to the tumor bed [14,36]. In the analyzed cohort, most patients received doses within the standard treatment range.

However, the possibility of administering a higher dose is associated with concerns regarding treatment toxicity. A meta-analysis that included 487 patients with LARC treated with doses exceeding 60 Gy demonstrated an early toxicity profile considered acceptable and a pCR rate of approximately 20% [37]. Furthermore, the use of modern radiotherapy techniques, such as intensity-modulated radiotherapy (IMRT), can help reduce toxicity without compromising treatment efficacy [38,39].

In our study, the finding that higher radiotherapy doses (>46 Gy) were independent predictors of improved tumor regression may support dose-escalation strategies in carefully selected patients, although this approach remains controversial in randomized trials.

A smaller tumor size has frequently been reported in the literature as being associated with a higher likelihood of achieving a pCR [24,25,26,27,40]. Furthermore, a reduction in tumor size to approximately 2–3 cm is an important criterion for eligibility for local excision following neoadjuvant chemoradiotherapy [26,27,41].

Contrary to these observations, in our cohort, tumor size was not significantly associated with response to neoadjuvant therapy. This result suggests that tumor regression is influenced to a greater extent by biological and therapeutic factors than by initial tumor size.

Numerous studies have highlighted the importance of the interval between the completion of neoadjuvant therapy and surgery for tumor regression [22,23,42]. Analysis of large cohorts of patients with LARC has demonstrated an increase in pCR rates when surgery is performed >7–8 weeks after completion of neoadjuvant therapy [29,41,43]. However, other studies have not confirmed the additional benefits of delaying surgery [44,45].

The results of our study support the hypothesis that a longer interval between radiotherapy and surgery may allow for the continuation of radiation-induced cell death and stromal remodeling processes, leading to progressive tumor regression and more favorable TRG scores.

Carcinoembryonic antigen (CEA) is a tumor marker frequently used in both the diagnosis and monitoring of colorectal cancer. Numerous studies have examined its predictive value for tumor response to neoadjuvant therapy [24,25,26,27,46]. For example, Wallin et al. demonstrated that low CEA levels are significantly associated with achieving pCR [24], an observation subsequently confirmed by other clinical studies [47].

In our analysis, patients with a favorable tumor response had significantly lower median CEA levels compared to patients without a favorable response, suggesting that pretreatment CEA levels may indirectly reflect tumor burden and the biological aggressiveness of the disease.

Systemic inflammation plays an important role in the progression of malignant tumors. The neutrophil-to-lymphocyte ratio (NLR) serves as an indirect indicator of the balance between systemic inflammation and the antitumor immune response. The study by Lee et al. demonstrated a correlation between NLR and the likelihood of tumor downstaging or achieving pCR after neoadjuvant therapy in rectal cancer [48]. In our cohort, the absolute lymphocyte count was independently associated with favorable tumor regression, suggesting that host immune status may influence the efficacy of chemoradiotherapy.

The influence of age on response to neoadjuvant therapy remains controversial. Some studies have suggested that younger patients have tumors with more aggressive biological characteristics and lower pCR rates [49,50], while other analyses have not identified significant differences between age groups [51]. In our analysis, age was not associated with the likelihood of tumor regression.

Our study has several strengths, including the analysis of a homogeneous cohort of patients with locally advanced rectal cancer and the use of multivariate analysis to evaluate predictors of tumor regression. However, there are also a number of limitations, including the retrospective design, the relatively small size of the subgroup of patients treated with TNT, and the possibility of unassessed confounding factors. Validation of these results in larger multicenter cohorts could help to strengthen the conclusions presented.

## 5. Conclusions

The results of this study highlight that the tumor response to neoadjuvant therapy in locally advanced rectal cancer is determined by a complex interaction between tumor biological characteristics, the host’s immune response, and the therapeutic parameters used. The complete pathological response rate observed in the analyzed cohort is comparable to that reported in the literature for standard neoadjuvant chemoradiotherapy, confirming the central role of this strategy in the management of LARC.

Our analysis suggests that both systemic biological factors and therapeutic variables may influence the likelihood of tumor regression. Biological markers, such as CEA and systemic inflammatory parameters, may reflect tumor burden and host immune status, contributing to the observed differences in treatment response. At the same time, therapeutic factors such as the neoadjuvant strategy used, the dose of radiotherapy, and the interval between the completion of treatment and surgery can have a significant impact on the likelihood of achieving a favorable tumor response.

Overall, the results of this study highlight the need to develop integrated predictive models that combine clinical, biological, and therapeutic parameters to enable more precise risk stratification. Such an approach could facilitate the individualization of therapeutic strategies and contribute to improving long-term oncological outcomes in patients with locally advanced rectal cancer.

## Figures and Tables

**Table 1:** Demographic data of categorical variables.

Variable	Category	*n*
Demographics		
Sex	Male	81 (66.4%)
	Female	41 (33.6%)
Smoking Status	Non-smoker	75 (61.5%)
	Smoker	47 (38.5%)
Obesity	No	95 (77.9%)
	Yes	27 (22.1%)
Tumor Characteristics		
Tumor Location	Inferior Rectum (RI)	88 (72.1%)
	Middle Rectum (RM)	27 (22.1%)
	Superior Rectum (RS)	7 (5.7%)
Histological Type	Adenocarcinoma (NOS)	117 (95.9%)
	Mucinous Adenocarcinoma	5 (4.1%)
Grading	G1	12 (9.8%)
	G2	107 (87.7%)
	G3	3 (2.5%)
Clinical Stage		
cTNM Stage	Stage II	7 (5.7%)
	Stage III	115 (94.3%)
Clinical T (cT)	T2	1 (0.8%)
	T3	104 (85.2%)
	T4	17 (13.9%)
Clinical N (cN)	N0	8 (6.6%)
	N1	66 (54.1%)
	N2	48 (39.3%)
Treatment and Response		
Chemotherapy Type	Capecitabine	82 (67.2%)
	TNT	40 (32.8%)
Radiation Dose	<46 Gy	20 (16.4%)
	>46 Gy	102 (83.6%)
Type of Surgery	Classical	64 (52.5%)
	Laparoscopic	58 (47.5%)
Ryan Score	0 (Complete response)	21 (17.2%)
	1	24 (19.7%)
	2	58 (47.5%)
	3 (Poor response)	19 (15.6%)
Pathological T (ypT)	ypT0	21 (17.2%)
	ypT1	11 (9.0%)
	ypT2	40 (32.8%)
	ypT3	44 (36.1%)
	ypT4	6 (4.9%)
Pathological N (ypN)	ypN0	77 (63.1%)
	ypN1	32 (26.2%)
	ypN2	13 (10.7%)

**Table 2:** Demographic data of continuous variables.

Variable	Mean	SD	Median	IQR
Age at diagnosis (years)	63.47	9.39	64.5	12
Longitudinal tumor dimension (mm)	47.06	19.8	45	26.5
Lymphocytes (10^3^/μL)	1.17	1.23	1	0.56
Neutrophils (10^3^/μL)	4.39	1.74	4.12	2.1
NLR	4.89	2.99	4.2	3.03
CEA (ng/mL)	5.18	4.97	3.5	4.56
Duration of RT (days)	41.31	13.68	39	9.75
Interval: End of RT to Surgery (days)	63.35	21.29	61.5	20.75
Distance to Anal Verge (mm)	69.89	23.75	60	30

**Table 3:** Differences between response groups.

Variable	Good Response (Ryan 0, 1) Mean (*n* = 45)	Poor Response (Ryan 2, 3) Mean (*n* = 77)	Test Statistic	*p*-Value
Age at diagnosis (years)	63.56	63.42	t = 0.079	0.937 ^a^
Longitudinal tumor dimension (mm)	46.4	47.44	U = 1701.0	0.869 ^b^
Preoperative lymphocyte count (10^3^/μL)	1.53	0.95	U = 2286.0	0.003 ^b^
Neutrophils (10^3^/μL)	4.34	4.41	U = 1661.0	0.706 ^b^
NLR	3.87	5.49	U = 1263.0	0.013 ^b^
CEA (ng/mL)	3.44	6.19	U = 1120.5	0.001 ^b^
Duration of RT (days)	41.09	41.44	U = 1857.0	0.509 ^b^
End of RT to Surgery (days)	72.73	57.87	U = 2455.0	<0.001 ^b^
Distance to Anal Verge (mm)	68.02	70.97	U = 1521.5	0.260 ^b^

^a^ = Student’s *t*-test, ^b^ = Mann–Whitney U-test.

**Table 4:** Simple logistic regressions predicting treatment response.

Predictor	Odds Ratio (OR)	95% CI for OR	Wald *p*-Value
Radiation Dose (>46 Gy)	0.152	0.034–0.692	0.015
Type of Chemotherapy (TNT)	0.135	0.058–0.315	<0.001
Type of Surgery (Laparoscopic)	0.797	0.382–1.665	0.546
Obesity (No)	0.819	0.333–2.018	0.665

For all models, the outcome was modeled as a binary event where a Ryan score of 2 or 3 (poor response) was coded as the target class (class 1).

**Table 5:** Exploratory multiple regression model.

Full Model Fit		McFadden R^2^: 0.417	*p* < 0.001
Predictor	Odds Ratio (OR)	95% CI for OR	Wald *p*-Value
Lymphocytes (Continuous)	0.263	0.076–0.910	0.035
NLR (Continuous)	1.258	0.959–1.651	0.098
End of RT to Surgery (Days)	0.963	0.930–0.998	0.037
CEA (Continuous)	1.263	1.035–1.542	0.022
Radiation Dose (>46 Gy)	0.086	0.010–0.747	0.026
Type of Chemotherapy (TNT)	0.083	0.025–0.276	<0.001

Outcome event (class 1) was defined as a Ryan score of 2 or 3 (poor response).

## Data Availability

The data presented in this study are available on request from the corresponding author. The data are not publicly available due to patient confidentiality.

## Abbreviations

The following abbreviations are used in this manuscript:

cCR	Clinical complete response
CEA	Carcinoembryonic Antigen
cN	Clinica Nodal Stage
cT	Clinical Tumor Stage
CT	Computed Tomography
cTNM	Clinical Tumor-Node-Metastasis Staging
CRM	Circumferential Resection Margin
CRT	Chemoradiotherapy
DFS	Disease-Free Survival
EMVI	Extramural Vascular Invasion
IMRT	Intensity-Modulated Radiation Therapy
LARC	Locally Advanced Rectal Cancer
LCRT	Long-course chemoradiotherapy
MRI	Magnetic Resonance Imaging
NCCN	National Comprehensive Cancer Network
NLR	Neutrophil-to-lymphocyte Ratio
NOS	Not Otherwise Specified
pCR	Pathological Complete Response
RT	Radiotherapy
SCRT	Short-course radiotherapy
TME	Total Mesorectal Excision
TNT	Total Neoadjuvant Therapy
TRG	Tumor Regression Grade

## Appendix A

**Table A1:** Ryan score distribution and type of chemotherapy.

				Type of Chemotherapy			
Ryan score				Capecitabine		TNT	
0				5.00		16.00	
				6.10 %		40.00 %	
1				13.00		11.00	
				15.85 %		27.50 %	
2				48.00		10.00	
				58.54 %		25.00 %	
3				16.00		3.00	
				19.51 %		7.50 %	
Total				82.00		40.00	
				100.00 %		100.00 %	

**Table A2:** Distribution of Ryan score according to clinical T stage.

		Ryan Score					
cT		0–1		2–3		Total	
2		1		0		1	
3		40		64		104	
4		4		13		17	
Total		45		77		122	

**Table A3:** Distribution of Ryan score according to clinical N stage.

		Ryan Score					
cN		0–1		2–3		Total	
0		3		5		8	
1		28		38		66	
2		14		34		48	
Total		45		77		122	

**Table A4:** Distribution of pathological tumor stage (ypT) according to chemotherapy regimen.

		Type of Chemotherapy					
ypT		Capecitabine		TNT		Total	
0		6		15		21	
1		4		7		11	
2		28		12		40	
3		39		5		44	
4		5		1		6	
Total		82		40		122	

**Table A5:** Distribution of pathological nodal stage (ypN) according to chemotherapy regimen.

		Type of Chemotherapy					
ypN		Capecitabine		TNT		Total	
0		45		32		77	
1		25		7		32	
2		12		1		13	
Total		82		40		122	
